# Genetic alterations, RNA expression profiling and DNA methylation of HMGB1 in malignancies

**DOI:** 10.1111/jcmm.17454

**Published:** 2022-06-28

**Authors:** Shoukai Yu, Lingmei Qian, Jun Ma

**Affiliations:** ^1^ Hongqiao International Institue of Medicine & Clinical Research Center Tongren Hospital Shanghai Jiao Tong University School of Medicine Shanghai China

**Keywords:** bioinformatics, COVID‐19 biomarker, expression, *HMGB1*, pan‐cancer

## Abstract

The high mobility group box 1 (HMGB1) is a potential biomarker and therapeutic target in various human diseases. However, a systematic, comprehensive pan‐cancer analysis of HMGB1 in human cancers remains to be reported. This study analysed the genetic alteration, RNA expression profiling and DNA methylation of HMGB1 in more than 30 types of tumours. It is worth noting that HMGB1 is overexpressed in malignant tissues, including lymphoid neoplasm diffuse large B‐cell lymphoma (DLBC), pancreatic adenocarcinoma (PAAD) and thymoma (THYM). Interestingly, there is a positive correlation between the high expression of HMGB1 and the high survival prognosis of THYM. Finally, this study comprehensively evaluates the genetic variation of *HMGB1* in human malignant tumours. As a prospective biomarker of COVID‐19, the role that *HMGB1* plays in THYM is highlighted.

## 1 INTRODUCTION

Some studies have recently recognized high mobility group box 1 (*HMGB1*) as a potential biomarker for severe COVID‐19.^1^, ^2^, ^3^, ^4^ The serum *HMGB1* of patients with severe COVID‐19 is significantly elevated. In some circumstances, exogenous *HMGB1* could promote the entry of SARS‐CoV‐2 into alveolar epithelial cells expressing the receptor *ACE2*.^2^ Genetic and pharmacological inhibition of the *HMGB1‐AGR* pathway can play an important role in blocking the expression of *ACE2*. *HMGB1* is a multifunctional protein that plays different roles in different cell compartments. Extracellular *HMGB1* is considered a damage‐associated molecular pattern (DAMP) protein in response to stress, which serves as the central mediator of lethal systemic inflammation in tissue injury or infection. Alarmins are constitutive endogenous molecules that are released and activate the immune system in the event of tissue injury.^5^, ^6^, ^7^ *HMGB1* is one of the prototypical alarmins that activate innate immunity.^8^ In addition, although the number of references to alarmins in the literature is increasing rapidly, the one most characteristic in health and disease is *HMGB1*. Finally, it is worth noting that cancer is known as one of the individual risk factors for COVID‐19, and many of the affected patients with COVID‐19 are patients with malignant tumours.^9^ During the current COVID‐19 outbreak, one of the potential risks for cancer patients is the limited ability to access to necessary medical services. Furthermore, patients with lung cancer who are ≥60 years of age tend to have higher risks for COVID‐19 infection.^9^, ^10^ However, comprehensive pan‐cancer analyses have yet to be conducted to investigate the potential impact of *HMGB1* aberration in human cancers. ^11^, ^12^

Here, we conducted a pan‐cancer analysis of *HMGB1* in malignant tumours. In the TCGA pan‐cancer analysis, the most common genetic alterations were investigated. Next, the expression of *HMGB1* in tumour tissues and normal control tissues was compared. Since the new COVID‐19 is mainly transmitted through the air, one focus should be respiratory tumours. Furthermore, this study studied the genetic disorders of *HMGB1* in cancer. Interestingly, COVID‐19 is related to aging and inflammatory diseases, and a dysfunctional thymus may be the predisposing factor. ^13^, ^14^ We report that *HMGB1* plays an important role in THYM. This result highlights the relationship between COVID‐19 patients and the disorders of the thymus gland through bioinformatics tools.

## 2 METHODS

### 2.1 Gene expression analysis of *HMGB1*

Initially, the tumour immune‐estimation resource, version 2 (TIMER2) webserver (http://timer.cistrome.org/) was used to investigate the mRNA expression difference of *HMGB1* between tumour and normal tissues for the different tumours derived from the TCGA project. However, there are specific tumours with no normal tissues or very limited normal tissues in the TCGA project. For these tumours, the GEPIA2 (http://gepia2.cancer‐pku.cn/, the gene expression profiling interactive analysis 2) webserver was used to compare box plots of the mRNA expression difference between the tumour tissues and the corresponding normal tissues of the genotype‐tissue expression (GTEx) database.^15^

To determine the difference in *HGMB1* protein expression between tumour tissues and the normal tissues, analyses of protein expression were performed on the Clinical Proteomic Tumour Analysis Consortium (CPTAC) datasets using the UALCAN (http://ualcan.path.uab.edu).^16^ Six tumours were available: breast cancer, ovarian cancer, colon cancer, renal cell cancer, endometrial cancer and lung adenocarcinomas. The UALCAN is a comprehensive and interactive web resource for analysing cancer OMICS data, including TCGA, MET500 and CPTAC.^16^ Furthermore, this study investigated *HMGB1* expression at different pathological stages across cancer types using the GEPIA2 stage‐plot module. The cut‐off value was set to 50% to separate the groups into high‐ and low‐expression cohorts.

### 2.2 Survival prognosis of *HMGB1*

GEPIA2 was also used to perform custom statistical methods, such as survival analyses on a given dataset to obtain differentially expressed genes or isoforms dynamically. The survival‐map module in GEPIA2 was applied to generate plots for overall survival (OS) and disease‐free survival (DFS). The cut‐off value was 50% to separate the groups into high‐ and low‐expression cohorts. The log‐rank test was used for hypothesis testing. The comparison/survival module, *p*‐Values, *q*‐Values and Kaplan–Meier plots of Disease‐Free, Overall, Disease‐specific and Progression‐Free were obtained for TCGA cases. Statistical analyses were performed using the ‘survival’ package with R statistical software, version 4.0.5.

### 2.3 DNA methylation and genetic alteration analyses

The DNA methylation level of *HMGB1* was analysed using the methylation panel from the CGA module via UALCAN.^17^, ^18^ More than 30 tumours were available for the analyses.

The cBio Cancer Genomics Portal (cBioPortal, https://www.cbioportal.org/) is a user‐friendly and exploratory analysis tool for investigating multidimensional cancer genomic data sets.^1^, ^19^ Genetic alterations of *HMGB1* in pan‐cancer were explored by the cBioPortal. The results of the mutations, amplifications, profound deletions and Copy number alteration (CNA) were gathered. The schematic diagram of the three‐dimensional (3D) structure of *HGMB1* mutations was shown in a graphic panel in the mutations module.

### 2.4 Immune infiltration analysis of *HMGB1*

The Immune‐Estimation module of the TIMER2 webserver was used to explore the association between the level of *HMGB1* expression and the abundance of immune cells of CD8+ T‐cells and cancer‐associated fibroblasts. The TIMER, EPIC, MCP‐COUNTER, CIBERSORT, CIBERSORT‐ABS, QUANTISEQ and XCELL algorithms were applied to estimate immune infiltration. The results are demonstrated by both heatmap and scatter plots. In addition, the *p*‐values and partial correlation (partial_cor) values were calculated using the purity‐adjusted Spearman's test.

### 2.5 Gene‐related enrichment analysis

The STRING website (https://string‐db.org/) was applied to search *HMGB1* under the protein name section in *Homo sapiens* organism.^20^, ^21^ The main parameters under the settings panel were set by checking (evidence) for the meaning of network edges and (Experiments) for active interaction sources. In addition, we selected (low confidence [0.150]) for the minimum required interaction score and (no more than 50 interactors) for the maximum number of interactors. Using these settings, 50 top *HGMB1*‐binding proteins were identified for further analysis.

The ‘Similar Gene Detection’ panel on the GEPIA2 webserver was applied to obtain the top 100 *HGMB1*‐correlated targeting genes based on the datasets from all TCGA tumours and normal tissues. Pearson correlation analysis of selected genes was conducted using the ‘Correlation Analysis’ module. The p‐value and the correlation coefficient were provided. TIMER2 produced the heatmaps; these contain the p‐values and partial correlation in the purity‐adjusted Spearman's test. The intersection analysis of the *HMGB1*‐binding and interacted genes was completed using a Venn diagram. Finally, the enriched pathway analyses were analysed using ‘clusterProfiler’ in R statistical software, version 4.0.5, and the bubble plots were produced by ‘tidyr’ and ‘ggplot2’ packages.

## 3 RESULTS

### 3.1 HMGB1 is overexpressed in three tumours out of 33 tumours

**FIGURE 1:**
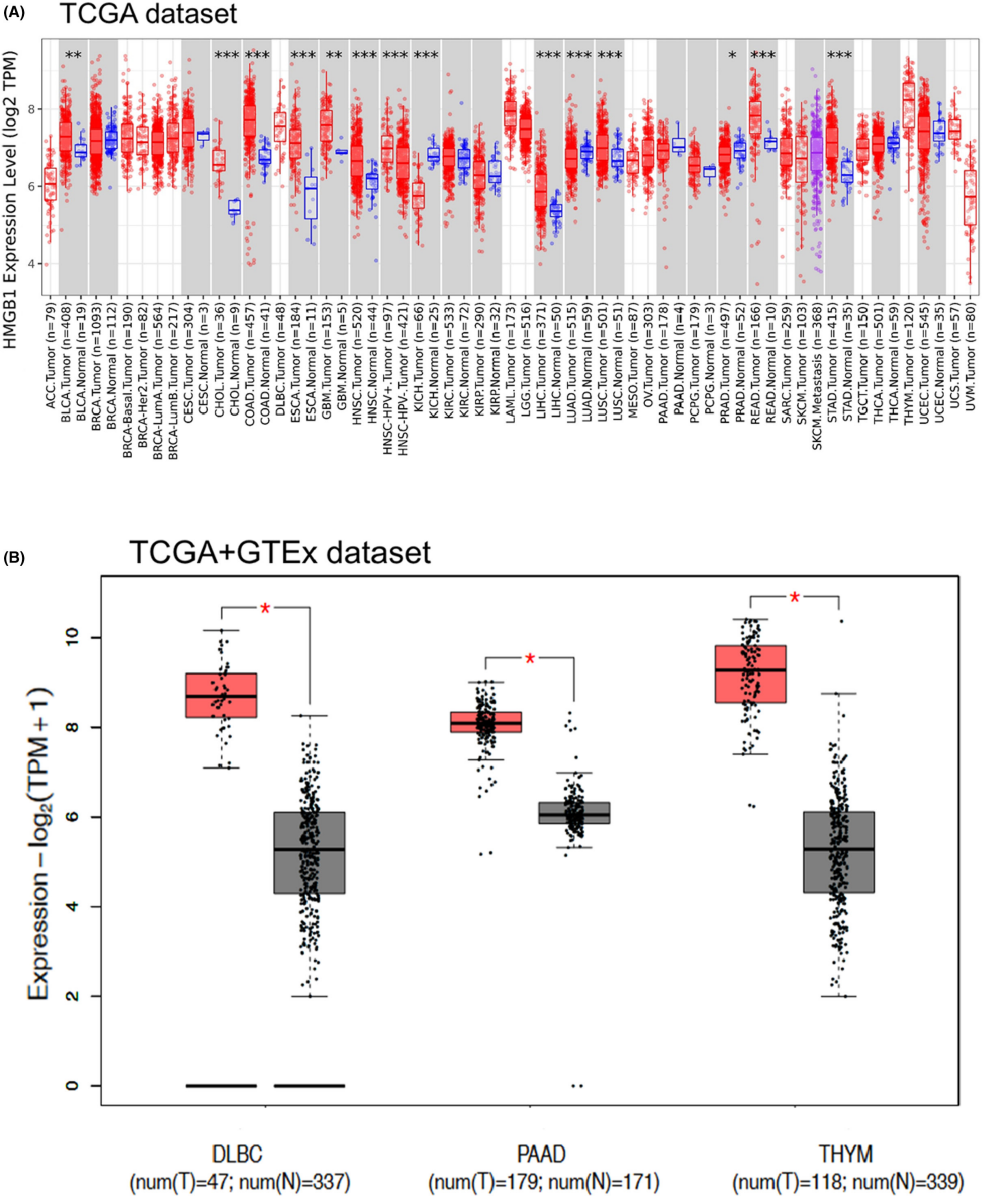
RNA expression of *HMGB1* in different tumours using TCGA and GTEx datasets. (A) The expression status of the *HMGB1* in different cancers or specific cancer subtypes was analysed through TIMER2. *p < 0.05; **p < 0.01; ***p < 0.001. (B) For the type of DLBC, PAAD and THYM in the TCGA project, the corresponding normal tissues of the GTEx database were included as controls. The red colour represents tumour groups, and grey represents normal controls

Initially, the expression pattern of *HMGB1* was analysed across various cancer types of TCGA using TIMER2. As shown in Figure 1A, the expression levels of *HMGB1* in the tumour tissues of CHOL (Cholangiocarcinoma), COAD (Colon adenocarcinoma), ESCA (Oesophageal carcinoma), HNSC (Head and neck squamous cell carcinoma), KICH (Kidney Chromophobe), LIHC (Liver hepatocellular carcinoma), LUAD (Lung adenocarcinoma), LUSC (Lung squamous cell carcinoma), READ (Rectum adenocarcinoma) and STAD (Stomach adenocarcinoma) are significantly different compared with the corresponding normal tissues (*p* < 0.001). Among them, CHOL, COAD, ESCA, HNSC, LIHC, LUSC, READ and STAD are significantly higher expressed in tumour groups, while KICH and LUAD are lower expressed in the tumour groups.

After combining the data from GTEx using GEPIA2 (Figure 1B), DLBC, PAAD and THYM presented the most significantly increased *HMGB1* expression (log2FC = 2 and *p* < 0.001, Figure 1B). Because COVID‐19 is mainly transmitted through the airway, we focused on respiratory system tumours, such as LUAD, LUSC and THYM. However, *HMGB1* remained unchanged in LUSC and only slightly increased in LUAD, the *p*‐value is not significant for LUAD.

The results of the CPTAC dataset showed lower expression of *HMGB1* total protein in the primary tissues of breast cancer, lung cancer and uterine corpus endometrial carcinoma (UCEC; Figure S1, *p* < 0.001) than in normal controls but not others. The ‘Pathological Stage Plot’ module of GEPIA2 was used to examine whether *HMGB1* expression may differ in different pathological stages of tumours. The outcomes indicated that *HMGB1* expression levels were significantly associated with the clinical stage of the following cancer types: Adrenocortical carcinoma (ACC) (*p*‐value = 0.0239), LIHC (*p*‐value = 0.0209), SKCM (*p*‐value = 0.0133) and THCA (*p*‐value = 0.0348) but not others (Figure S2).

### 3.2 Overexpression of *HMGB1* is linked to poor prognosis in five tumours

After examining the significant dysregulation of *HMBG1* expression in different cancer types and its correlation with the pathological stage, one potential hypothesis is that this protein might be used as a prognostic indicator for certain cancer types. The cancer samples were divided into high‐ and low‐expression groups based on the expression levels of *HMGB1*. Then, the associations between the expression level of *HMGB1* and prognostic significance with different tumours derived from TCGA and GEO databases were investigated.

**FIGURE 2:**
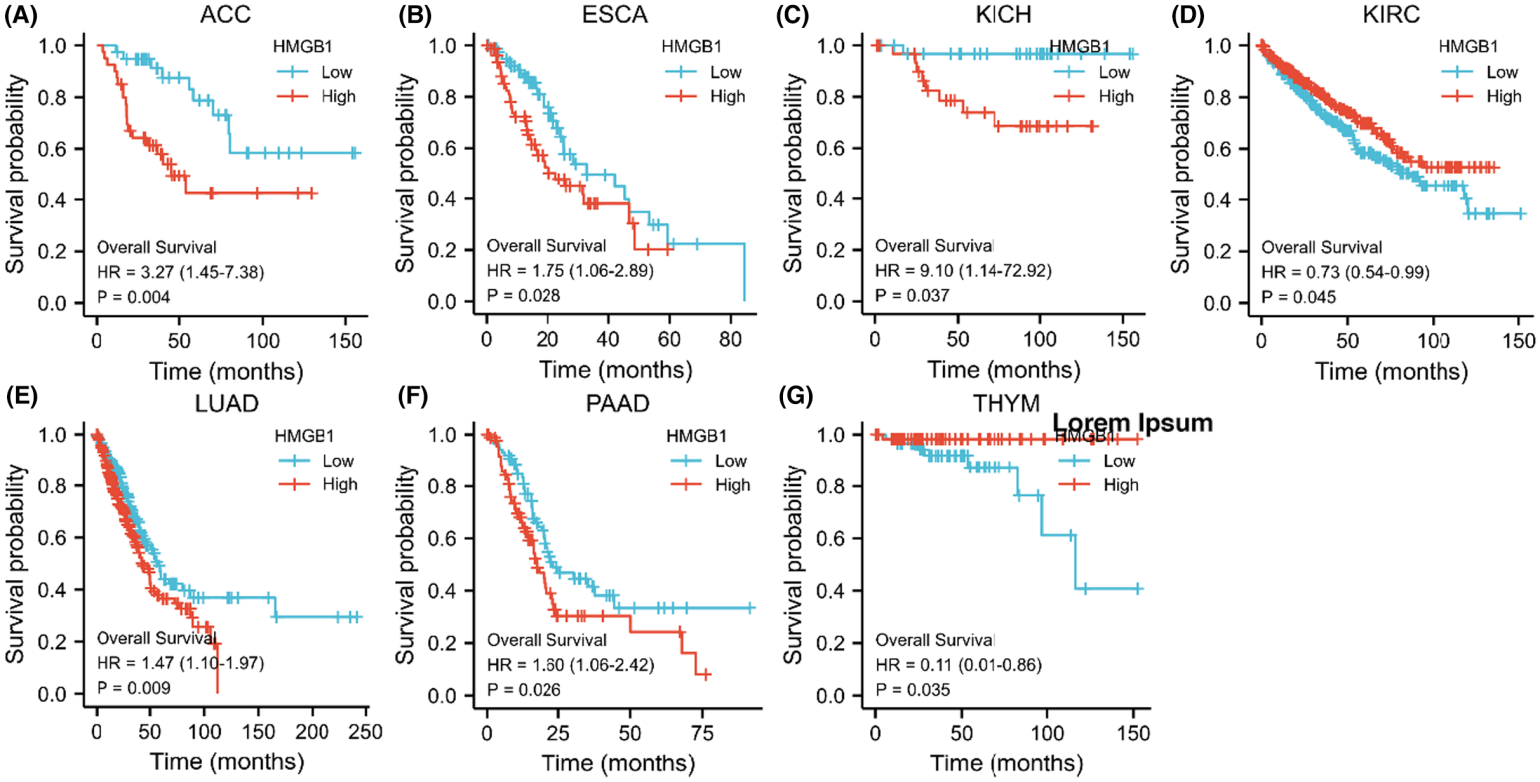
Overall survival (OS) data in *HMGB1* abnormally expressed malignancies. In the different malignancies, the OS data are not directly related to the expression levels of *HMGB1*

As shown in Figure 2, highly expressed *HMGB1* was significantly associated with Overall Survival (OS) for cancers of ACC (*p* = 0.004), ESCA (*p* = 0.028), KICH (*p* = 0.037), KIRC (*p* = 0.045), LUAD (*p* = 0.009), PAAD (*p* = 0.026) and THYM (*p* = 0.035). DFS analysis showed that high *HMGB1* expression is significantly correlated with poor prognosis for only HNSC (*p* = 0.025). These results indicated that the level of *HMGB1* expression is differentially associated with the prognosis of different cancer types. Three tumours (DLBC, PAAD and THYM) presented significantly elevated *HMGB1* expression. Both DFS and OS results demonstrated no direct relationship between *HMGB1* expression and patient prognosis.

This study showed that, compared with normal samples, the RNA expression of *HMGB1* was not significantly up‐ or down‐regulated in LUADs. However, both the OS and DFS results of *HMGB1* showed that the higher expression of *HMGB1* could lead to significantly poorer patient outcomes in LUAD (Figure S3). This may indicate that RNA expression of *HMGB1* is not correlated with patient outcomes. The DFS and OS are not significant for *HMGB1* in LUSC (Figure S3). The HMGB1 serves as a double‐edged sword for patients with different tumours. For example, for OS, higher *HMGB1* expression indicates a better prognosis in KIRC and THYM, but a significantly unfavourable outcome in ACC, ESCA, KICH, LUAD, PAAD and PRAD.

### 3.3 DNA methylation and genetic alteration analysis

Eleven probes in the *HMGB1* promoter were used in this study to detect the DNA methylation level of *HMGB1* (Figure S4). Interestingly, for respiratory system‐related tumours, such as LUAD, LUSC and THYM, the DNA methylation levels of *HMGB1* were all decreased. There are three tumours (DLBC, PAAD and THYM) with the higher mRNA expression levels of *HMGB1*. However, the DNA methylation level of *HMGB1* for these three tumours are not consistent. For example, PAAD with upregulated *HMGB1* presented a significantly decreased DNA methylation level. Conversely, one *HMGB1* upregulated tumour, THYM, presented a slightly upregulated DNA methylation level.

Furthermore, because there is no available DNA methylation dataset for DLBC normal control, the comparison analyses were conducted across different patient populations. Similarly, the comparison is not statistically significant for DLBC. These results confirmed that abnormal *HMGB1* expression was not solely due to DNA methylation. Further exploration should be done for histone modifications and glycosylation.^22^, ^23^

**FIGURE 3:**
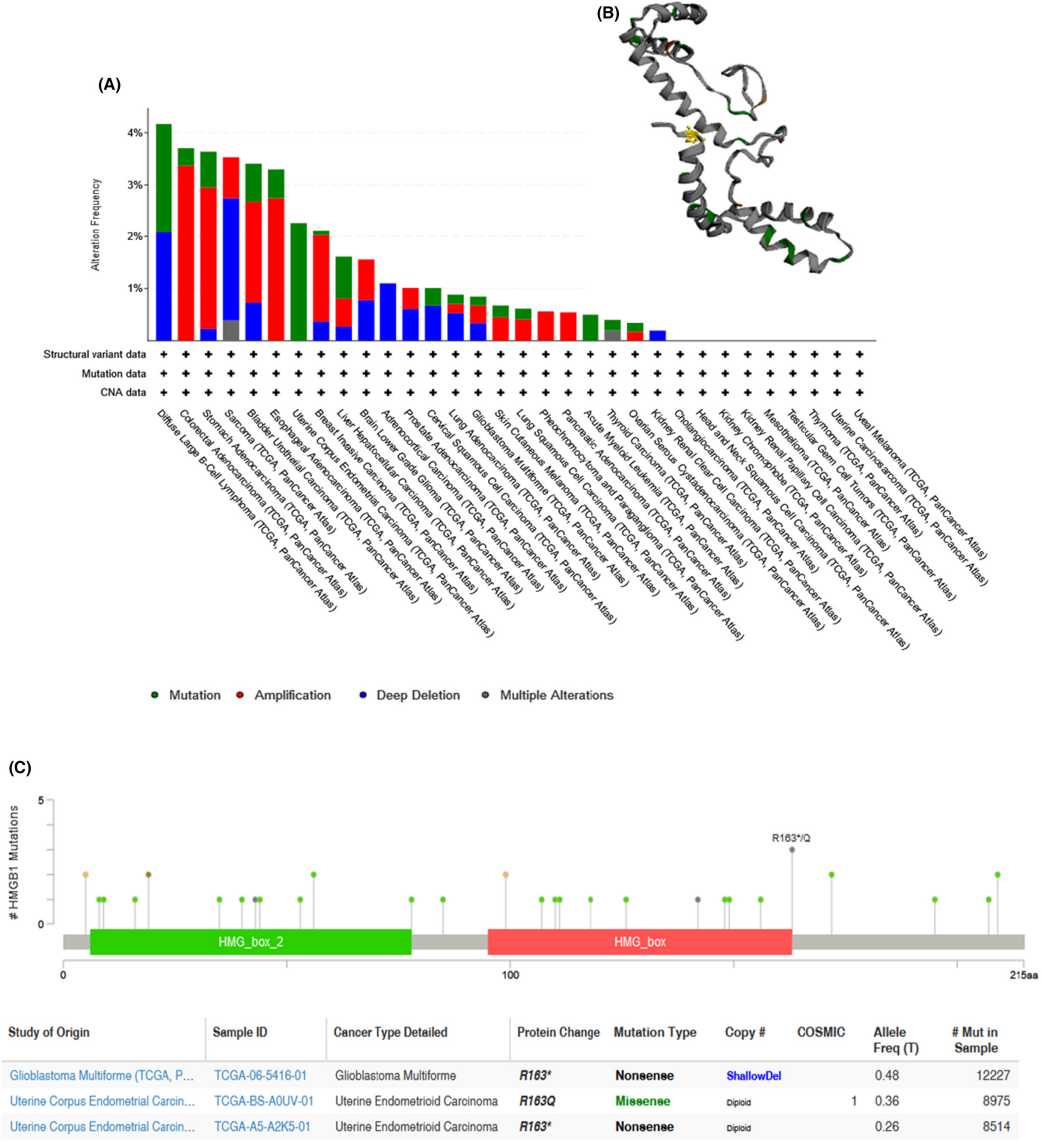
(A) Genetic variation of *HMGB1* in different tumours. (B) The 3D structure of HMGB1 mutations. The yellow colour highlighted the mutation R163*/Q. (C) *HMGB1* mutations were distributed across all exons of *HMGB1* without hot spot mutation site in TCGA cohort using cBioPortal

Genetic alterations of *HMGB1* were observed among different cancer samples from the TCGA database. The pan‐cancer analysis of *HMGB1* in different malignancies demonstrates that the most frequent DNA alterations are amplification, mutations and deep deletions in the TCGA pan‐cancer panel (Figure 3A). Amplification was mainly distributed in COAD, STAD, bladder urothelial carcinoma (BLCA) and ESCA. Mutations were mainly distributed in DLBC, UCEC and LIHC. The most frequent deep deletions were observed in DLBC, sarcoma (SARC) and ACC patients (Figure 3A). For DLBC, SARC and ACC patients, the deep deletions appeared more than 50% in alteration frequency. In addition, *HMGB1* mutations in different malignancies were distributed across HMG_box and HMG_box_2 domains without hot spot mutation sites (Figure 3C). The most observed frequent mutation was R163*/Q; the 3D structure of the *HMGB1* mutations is shown in a graphic panel (Figure 3C).

The results showed that mutations were not statistically relevant to RNA expression of *HMGB1* (Figure S5). Furthermore, copy variations were also not significantly relevant to *HMGB1* expression (Figure S5). One possible explanation is that the upregulation of *HMGB1* expression is not a direct consequence of genetic variation. Thus, we further investigated the post‐translation features of *HMGB1* in 33 cancers.

### 3.4 Phosphorylation levels of *HMGB1* in several cancers

The differences in *HMGB1* phosphorylation levels were compared between normal tissue and primary tumour tissues using CPTAC datasets for four types of tumours (breast cancer, clear cell carcinoma, LUAD and UCEC). Figure S6 summarizes the phosphorylation sites of *HMGB1*, which are significantly different from the control group: S35 locus and S100 locus. The S35 locus demonstrates a significantly lower phosphorylation level in primary tumour tissues compared with normal tissues for breast cancer (*p* = 2e‐05), LUAD (*p* = 6e‐38) and UCEC (*p* = 9e‐06) (Figure S6). By contrast, the S100 locus is the only one to exhibit a significantly decreased phosphorylation level for breast cancer (Figure S6, *p* = 2e‐04), but not for LUAD and UCEC.

### 3.5 Immune infiltration analysis

As an important part of the tumour microenvironment, tumour‐infiltrating immune cells were reported to be closely related to the initiation, promotion, progression or metastasis of tumours.^24^, ^25^ Furthermore, according to previous research, cancer‐associated fibroblasts regulate the functions of various cancer‐infiltrating immune cells.^26^, ^27^ Therefore, the algorithms of TIMER, CIBERSORT, CIBERSORT‐ABS, QUANTISEQ, XCELL, MCPCOUNTER and EPIC were used to study the potential relationship between the expression of *HMGB1* and the infiltration level of different immune cells in TCGA for different tumour types.

**FIGURE 4:**
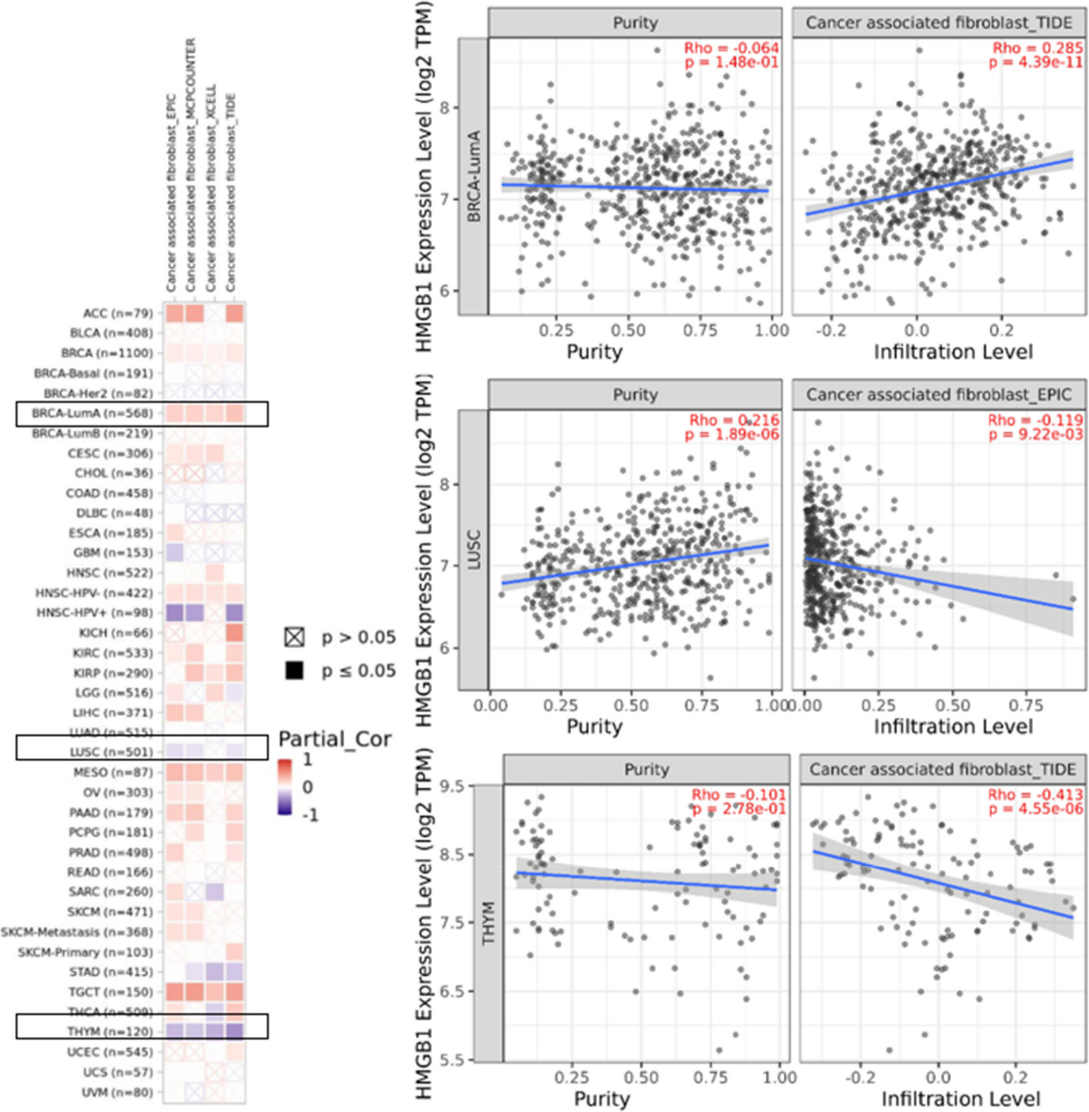
Relationship between *HMGB1* expression and cancer‐associated fibroblasts (CAFs). Four algorithms (EPIC, MCPCOUNTER, XCELL and TIDE) were used to investigate the possible relationship between *HMGB1* expression and infiltration of CAF in various cancer types. The right panel shows the correlation and scatterplot for the three selected cancer types

After a series of analyses, statistically positive correlations were observed between *HMGB1* expression and CD8+ T‐cell immune infiltration in HNSC‐HPV+, LUAD, LUSC and THYM based on seven out of the ten algorithms (Figure S7). These positive correlations do not infect the prognosis directly. In addition, the positive correlations were detected between *HMGB1* expression and the immune infiltration of cancer‐associated fibroblasts in the TCGA tumours of BRCA‐LumA, MESO and TGCT based on all or most algorithms (Figure S8). The negative correlations were detected between *HMGB1* expression and the immune infiltration of cancer‐associated fibroblasts in the TCGA tumours of HNSC_HPV+ based on all or most algorithms. The scatter plots of these tumours were also provided for one of the most significant algorithms. For example, the expression level of *HMGB1* in THYM is statistically positively correlated with the infiltration level of cancer‐associated fibroblasts (Figure 4, cor = −0.413, *p* = 4.55e‐06) based on the TIDE algorithm.

### 3.6 Enrichment of HMGB1‐related partners

**FIGURE 5:**
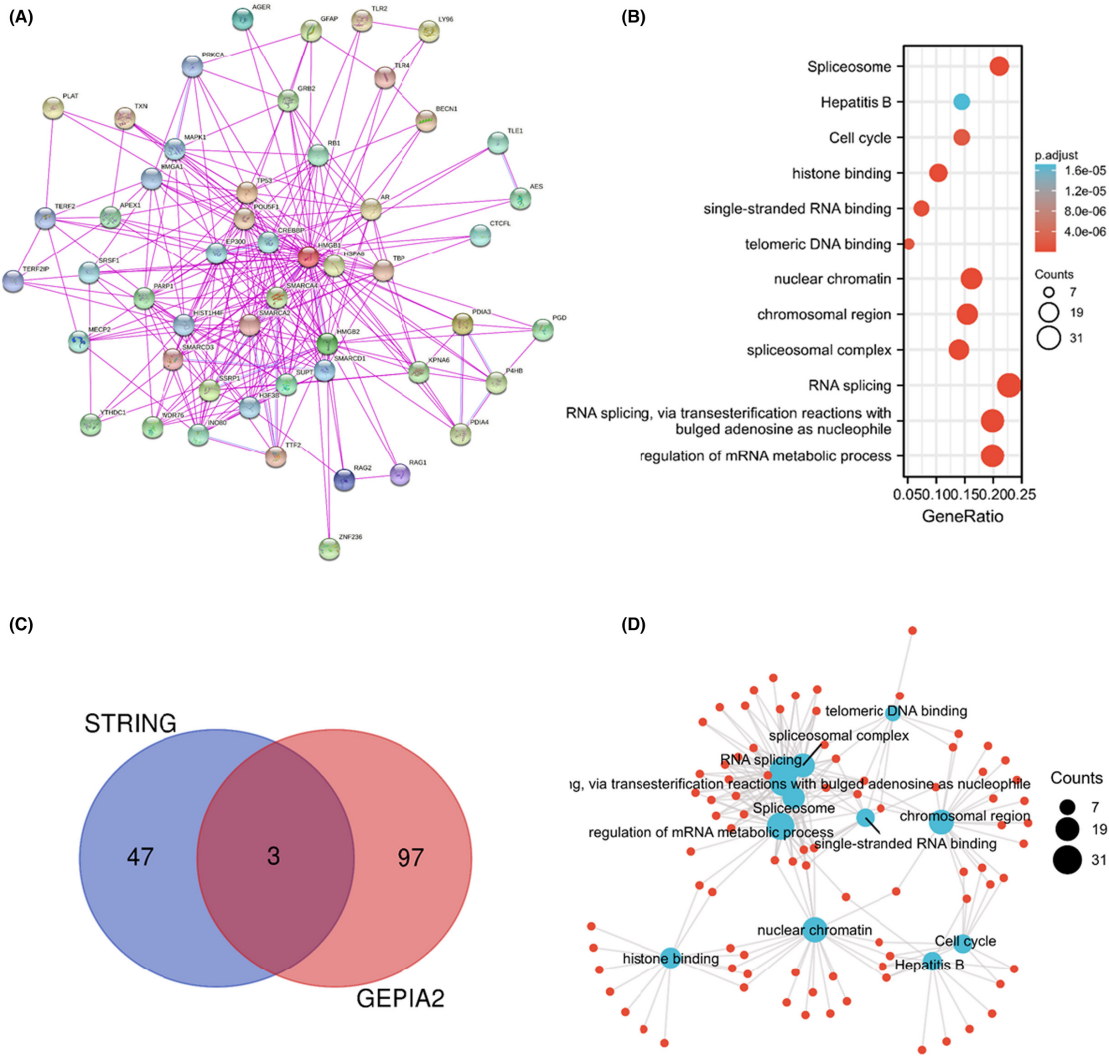
Enrichment analysis of the *HMGB1* gene. (A) Fifty proteins that bind to *HMGB1* were identified using the STRING tool. In addition, 100 genes associated with *HMGB1* were acquired from the TCGA database. (B) KEGG pathway analysis based on the *HMGB1*‐binding and interacted genes. (C) An intersection analysis of the *HMGB1*‐binding and correlated genes was conducted. (D) The cnetplot for the molecular function data in GO analysis

To further explore the molecular mechanism of *HMGB1* in tumorigenesis, the *HMGB1* expression‐related genes or proteins were obtained from a series of pathway enrichment analyses. First, 50 binding proteins were observed using the STRING tool, all supported by experimental evidence. The interaction network of these proteins is presented in Figure 5. Next, based on the GEPIA2 tool, the top 100 genes related to HMGB1 expression were obtained by combining all tumour expression data of TCGA. Finally, the two datasets were combined to perform further KEGG and GO enrichment analyses.

An intersection analysis of these two datasets contained three common members (HMGB2, SRSF1 and SSRP1). Moreover, the related heat maps demonstrated that there are positive correlations between *HMGB1* and RP11‐673C5.1 (R = 0.87), HMGB1P5 (R = 0.69), EXOSC8 (R = 0.69), HNRNPA2B1 (R = 0.68), SRSF3 (R = 0.67), MED4 (R = 0.65), RFC3 (R = 0.63) and HNRNPR (R = 0.63). These positive correlations are statistically significant (all P < 0.001). In addition, the corresponding heat map demonstrates that in most cancer types, there is a positive correlation between *HMGB1* and the above five genes (Figure S9).

The two data sets (obtained by STRING and GEPIA2) were combined for further KEGG and GO enrichment analysis. The KEGG and GO results highlight the following potential pathways: ‘RNA splicing’, ‘RNA splicing, via transesterification reactions with bulged adenosine as a nucleophile’ and ‘regulation of mRNA metabolic process’ in biological processes (BP) GO components and ‘Spliceosome’ in the KEGG pathway database (Figure 5).

## 4 DISCUSSION

Emerging applications report the functional link between *HMGB1* and clinical diseases, especially COVID‐19.^1^, ^2^, ^3^, ^4^ However, the role of the multifunctional *HMGB1* in the molecular pathogenesis of different tumours remains unclear. This study analysed the genetic changes, RNA expression, protein expression and DNA methylation of *HMGB1* in more than 30 tumours. A significant overexpression of *HMGB1* was observed in DLBC, PAAD and THYM. The correlation analysis of *HMGB1* and survival prognosis has also been performed. In addition, low DNA methylation of *HMGB1* was found in most tumours with high *HMGB1* expression. The result of genetic alterations (Figure 3A) demonstrates that the most frequent DNA alterations are amplification, mutations and deep deletions. The patterns of genetic alterations for *HMGB1* differ across cancer types. For DLBC, both the mutations and deep deletions were observed in DLBC patients. For SARC, the most frequent genetic alterations are deep deletions. In conclusion, this study investigates the genetic variation of *HMGB1* in human malignant tumours.

LUAD is the most common type among the COVID‐19 patients with malignant tumours.^28^, ^29^ In addition, lung cancer patients have been confirmed to have a higher COVID‐19 incidence and more severe symptoms.^28^, ^29^ Here, we demonstrated that RNA expression of HMGB1 is significantly upregulated in THYM patients but not significantly changed in LUAD and LUSC. The phosphorylation analyses using the CPTAC dataset included four cancer types. Results demonstrated the decreased phosphorylation levels of S35 and S100 for different tumours. Furthermore, the findings showed that compared with the normal control group, the total protein and phosphorylation level of HMGB1 at the S35 locus in the primary tumour was significantly lower for breast cancer, LUAD and UCEC (Figure S6, all *p* < 0.01). However, the total protein levels of *HMGB1* were significantly higher for both ovarian cancer and colon cancer. Although the clinical significance of these post‐translational modification sites remains to be determined, the current analyses do not rule out the possibility that the significantly decreased level of *HMGB1* phosphorylation of S35 is a by‐product of a functionally significant dysregulated signal in tumour cells. In addition, more experiments are needed to evaluate further the potential role of S35 and S100 phosphorylation of *HMGB1* and the role of related cell cycle regulation in tumorigenesis.

The significant changes of phosphorylation are consistent with the expression level of *HMGB1* total protein between normal tissue and primary tissue for breast cancer, clear cell RCC and UCEC (Figure S1 and Figure S6). The change of phosphorylation is not directly correlated with expression of *HMGB1* (Figure 1 and Figure S6). Moreover, there are statistically positive correlations observed between *HMGB1* expression and CD8+ T‐cell immune infiltration in HNSC‐HPV+, LUAD, LUSC and THYM, however, these positive correlations do not infect the prognosis directly (Figure 2 and Figure S7). Interestingly, the high expression of *HMGB1* is related to the significantly increased survival rate for THYM, based on the OS result for *HMGB1* (Figure 2), which may indicate the potential function of HMGB1 in specific tumours.

As this is a pan‐cancer analysis and the presented results show that the function of *HMGB1* is different in different cancer types, and the relationship of prognosis are different from the results of immune infiltrations. These results demonstrated that the function of *HMGB1* in different cancer types is different, such as the correlation of *HMGB1* with CAFs is positive in BRCA‐LumA, MESO and TGCT; but is negative in HNSC‐HPV+, which may indicate that the mechanism of *HMGB1* is different in different cancer types.

*HMGB1* is quickly released into the circulation in severe mechanical trauma, related conditions and sepsis.^3^, ^30^ This is related to the destructive and self‐harming features of the innate immune response. In some life‐threatening diseases, *HMGB1* levels are remarkably high and associated with acute inflammation, such as stroke and acute myocardial infarction.^3^, ^30^ In the most severely ill patients, *HMGB1* autoantibodies in sepsis models are associated with a good prognosis.^31^ In injury‐mediated sterile inflammation, *HMGB1* is released as an early mediator to activate the release of TNF‐α and other cytokines. In animals, systemic administration of *HMGB1* could be fatal.^32^ Many animal studies have shown the beneficial use of neutralizing antibodies or recombinant antagonists to inhibit HMGB1, thrombomodulin box A or the N‐terminal portion on haemorrhagic shock,^33^, ^34^ ischemia/reperfusion,^35^ myocardial^36^ and acute lung.^37^ In contrast, *HMGB1* could also serve as an advanced mediator of sepsis and have beneficial effects in preclinical sepsis studies.^38^, ^39^

A wide range of immune deconvolution methods were applied to investigate the correlation between *HMGB1* expression and the immune infiltration level of CD8+ T‐cells in 33 tumours. The results first suggested the correlation between HMGB1 expression and the estimated infiltration value of cancer‐associated fibroblasts in certain tumours, including the TCGA tumours of BRCA‐LumA, HNSC_HPV‐, MESO and TGCT. The DNA methylation levels were down‐regulated for LUAD, LUSC and THYM (Figure S4). There are positive correlations between *HMGB1* expression and the immune infiltration level of CD8+ T‐cells in lung and thymic cancers, such as LUAD, LUSC and THYM (Figure S7). It is worth noting that the current research is based on bioinformatics analysis. Therefore, further functional and clinical verification is necessary.

Interestingly, the mRNA expression of *HMGB1* is significantly increased in THYM (Figure 1B), and this increased expression could lead to a better OS for patients with THYM (Figure 2). Different from other cancer types, there is a significantly negative correlation between THYM and cancer‐associated fibroblasts (Figure S8). At the same time, there is a significantly positive correlation between THYM and T‐cell CD8+ (Figure S7). Furthermore, the results from STRING and GEPIA2 analyses shared three members (*HMGB2, SRSF1* and *SSRP1*) for the enrichment analyses of *HMGB1*‐related partners (Figure 5C, Figure S10). These three members have been reported to be associated with lung cancers or breast cancers.^40^, ^41^, ^42^, ^43^, ^44^, ^45^

In this study, we unified several publicly available databases to investigate the expression of the *HMGB1*, explored correlations with prognosis and evaluated potential mechanisms of regulation in tumour patients. We utilized the TCGA, ONCOMINE, cBioPortal, UALCAN, GEPIA and STRING databases to obtain a comprehensive understanding of the structure and function of the *HMGB1*. Based on the results of the correlation analysis of *HMGB1* and survival prognosis using GEPIA2, it can be seen that the overexpression of *HMGB1* is significantly associated with poor prognosis of the five tumours (ACC, ESCA, KICH, LUAD and PAAD), while the overexpression of *HMGB1* is also significantly associated with better prognosis of KIRC and THYM. These results suggest that the expression of *HMGB1* has the potential to serve as a poor prognostic biomarker and therapeutic target for cancer patients.

COVID‐19 is a respiratory disease that causes severe symptoms in the lungs. However, one of the differences from other respiratory diseases is that the high fatality rate is initially due to thick, copious mucus in the lungs and then, to the impairment of lung function ^46^, ^47^. Therefore, the disease of the chest cavity caused by thymic cancer, such as THYM, could greatly promote mucus secretion or make it easier for the mucus to affect the lungs. This finding helps us understand the further impact of thoracic cavity structure and function on COVID‐19, rather than only focusing on lung function. To the best of our knowledge, this is the first discovery of COVID‐19 and THYM through *HMGB1*.

In summary, the pan‐cancer analysis of *HMGB1* showed that the expression of *HMGB1* was significantly related to the prognosis, genetic changes, immune cell infiltration and drug sensitivity of different tumours in cancer patients. *HMGB1* acts as a tumour promoter in most of the tumours studied and has the potential to be used as a potential marker for prognosis. This helps us understand the role of *HMGB1* in tumorigenesis. Most conclusions were drawn from bioinformatics analysis in the current research. More experiments are needed to evaluate further the potential role of HMGB1 in THYM to support the bioinformatic results. Thus, further research should explore how HMGB1 promotes tumorigenesis, such as through the analysis of gene alterations and the related signalling pathways. For cancer treatment, further research should pay attention to the role of *HMGB1* in immunotherapy and targeted therapy.

## AUTHOR CONTRIBUTIONS

**Shoukai Yu:** Conceptualization (lead); formal analysis (equal); methodology (equal). **Lingmei Qian:** Investigation (equal). **Jun Ma:** Conceptualization (supporting); formal analysis (equal); investigation (equal).

## CONFLICT OF INTEREST

The authors confirm that there are no conflicts of interest.

## DATA AVAILBILITY STATEMENT

All the data used in this study are obtained from publicly available databases, the data and results analysed in this study are available on request.

## Supporting information


Figure S1
