# Supplementary material for: Genetic alterations, RNA expression profiling and DNA methylation of HMGB1 in malignancies
Source: J Cell Mol Med. 2022 Jun 28;26(15):4322–32. doi: 10.1111/jcmm.17454 (PMC9344825; doi:10.1111/jcmm.17454)
Supplement: Supplementary file 1 — Figure S1 [file JCMM-26-4322-s001.docx]

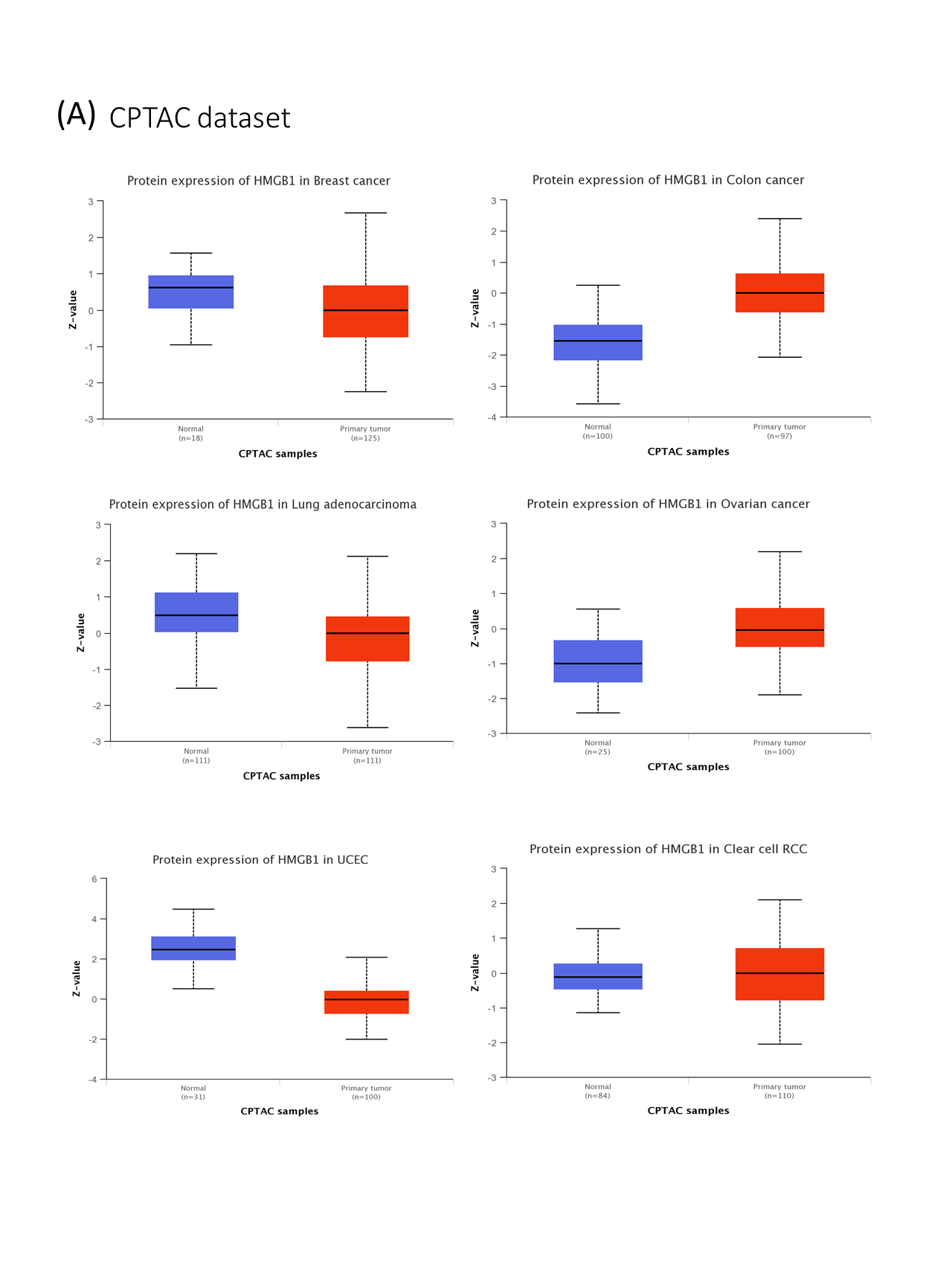


**Figure S1.** Expression of *HMGB1* in different tumors. Based on the CPTAC dataset, we also analyzed the expression level of *HMGB1* total protein between normal tissue and primary tissue of breast cancer, ovarian cancer, colon cancer, clear cell RCC, and UCEC. ***p < 0.001.


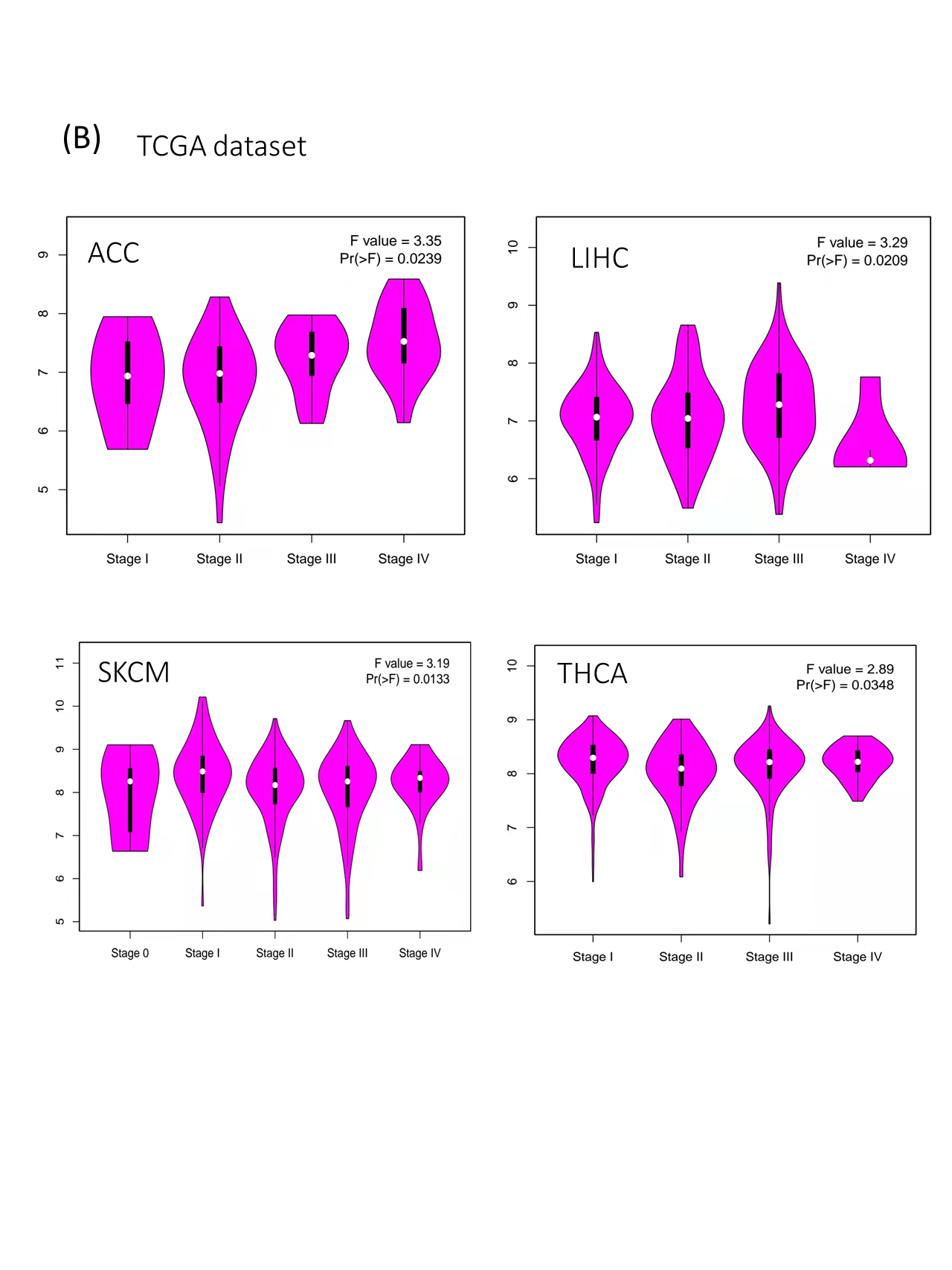


**Figure S2.** Based on TCGA data, the expression levels of *HMGB1* were analyzed by the main pathological stages of ACC, LIHC, SKCM, and THCA. A Log2 (TPM +1) transformation was used for the log scale. Y-axis represents the expression of *HMGB1* Log2 (TPM +1).


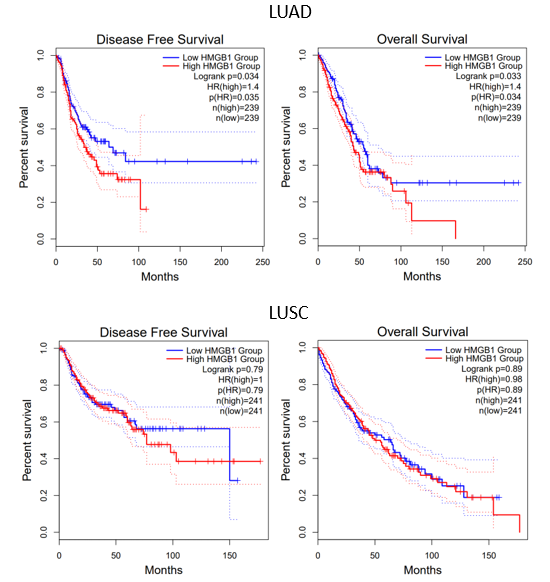


**Figure S3.** Disease-free survival (DFS) data and overall survival (OS) data for *HMGB1* in LUAD and LUSC.


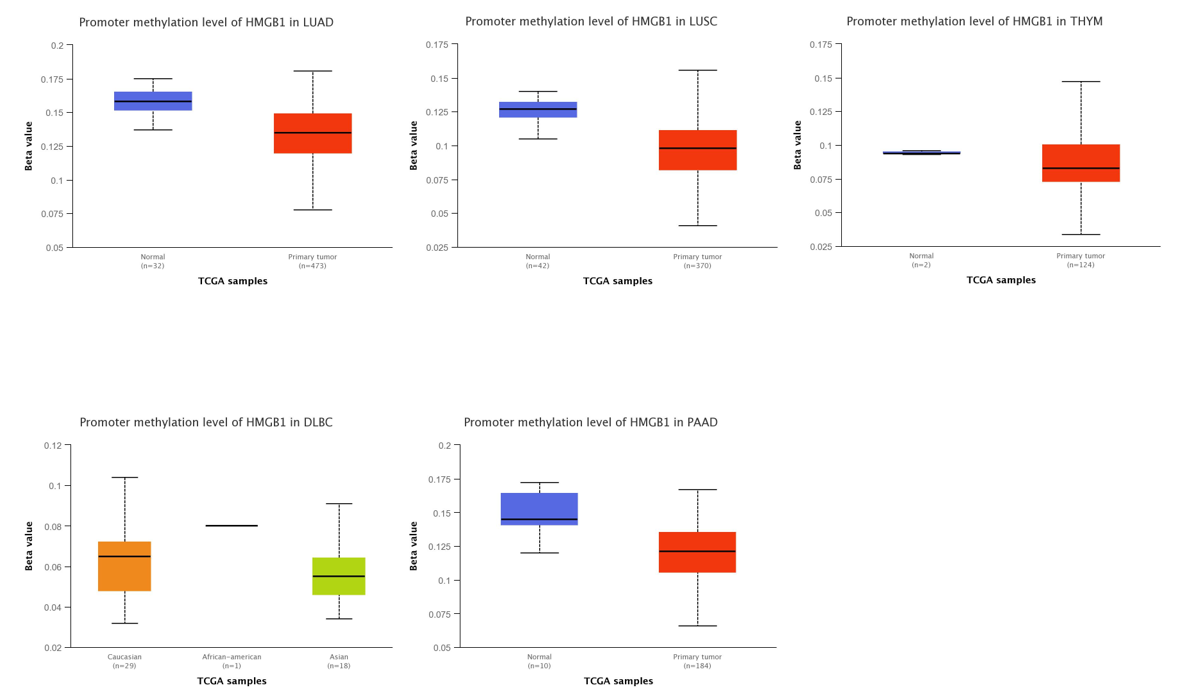


**Figure S4.** DNA methylation level of *HMGB1.*

+

**Figure S5.** Relevance of genetic disorders and *HMGB1* expression. (A) Mutations were not relevant to RNA expression. (B) DNA copy variations were not statistically relevant to the RNA expression of *HMGB1* in most cases.


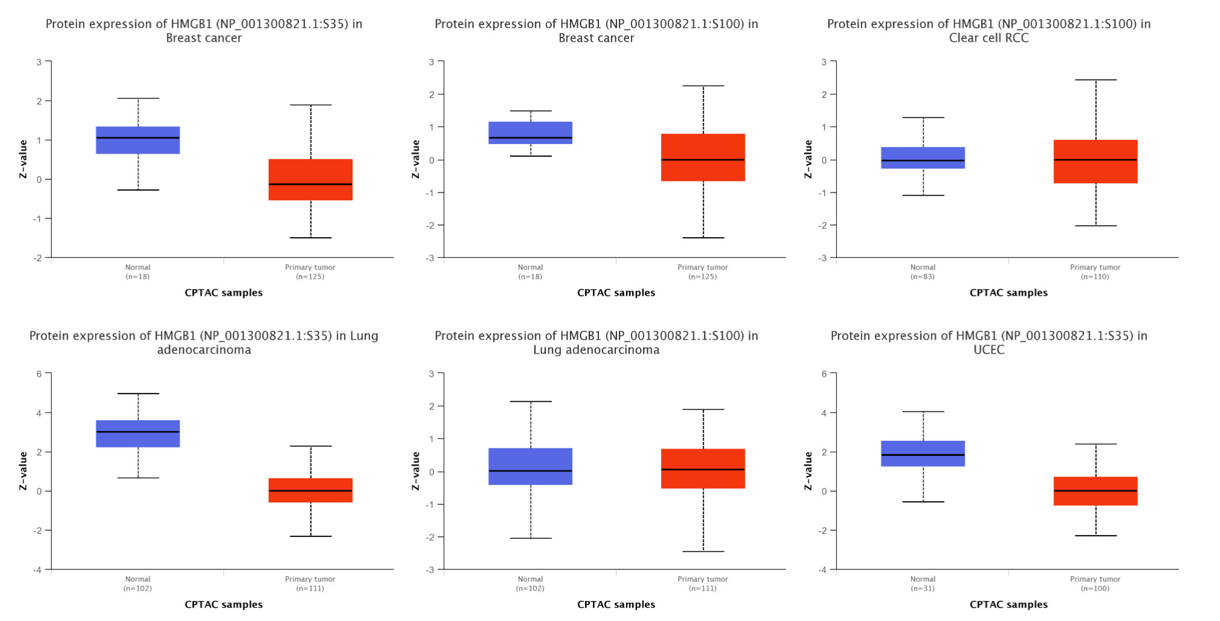


**Figure S6.** Phosphorylation analysis of *HMGB1* in different tumors. Based on the CPTAC dataset, the protein expression of *HMGB1* was analyzed between the normal tissue and primary tissue of selected tumors via the UALCAN.


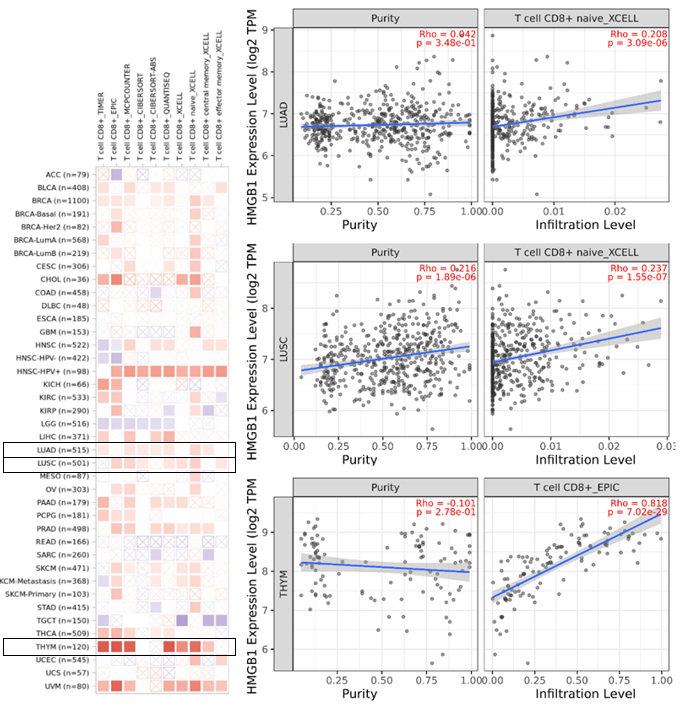


**Figure S7.** Relationship between *HMGB1* expression and immune infiltration of CD8+ T cells. Ten algorithms were used to investigate the possible relationship between *HMGB1* expression and infiltration of CD8+ T cells in various cancer types.


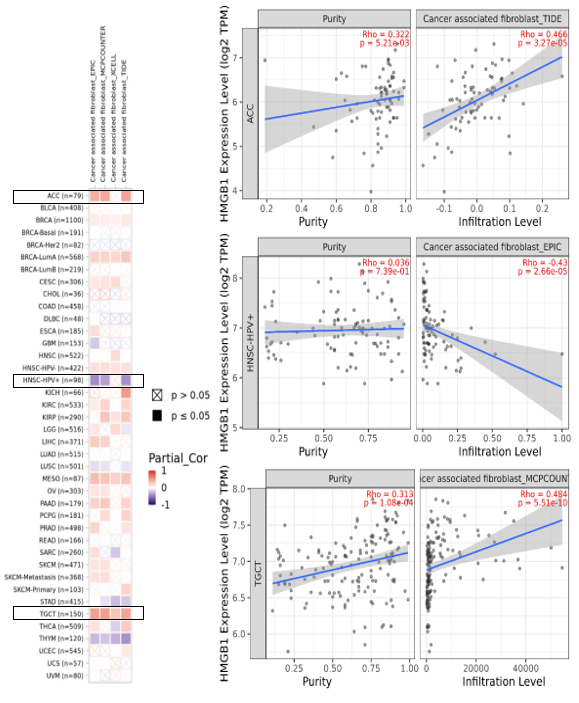


**Figure S8.** Relationship between *HMGB1* expression and cancer-associated fibroblasts (CAFs). Four algorithms (EPIC, MCPCOUNTER, XCELL, and TIDE) were used to investigate the possible relationship between *HMGB1* expression and infiltration of cancer-associated fibroblasts in various cancer types.


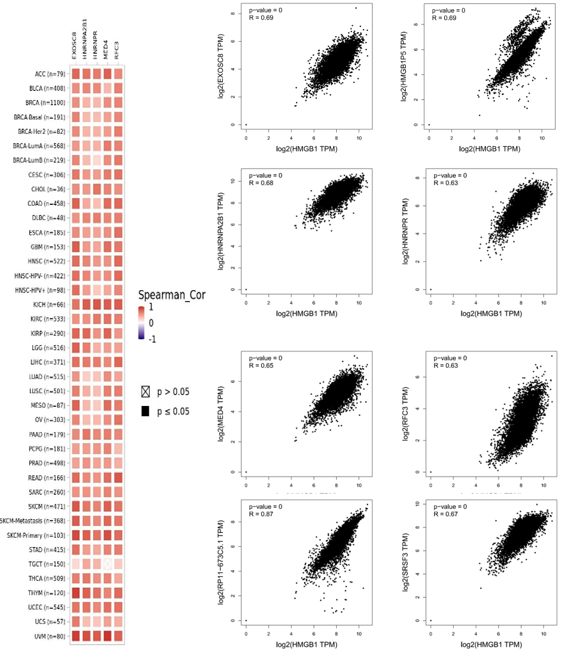


**Figure S9.** (Left) Heatmap data in the detailed cancer types are displayed. (Right) Expression correlation between *HMGB1* and the selected targeting genes was analyzed.


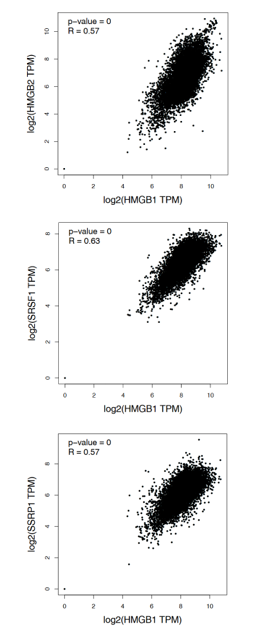


**Figure S10.** Expression correlation between *HMGB2, SRSF1, SSRP1*, and *HMGB1* was analyzed separately.
